# Factors related to the risk of developing chronic kidney disease in resistant hypertensive patients: a cross-sectional study

**DOI:** 10.1590/0034-7167-2025-0160

**Published:** 2026-06-12

**Authors:** Alessandra de Oliveira Guimarães, Nicole Oliveira Santana, Ronaldo Altenburg Gismondi, Dayse Mary da Silva Correia

**Affiliations:** I Universidade Federal Fluminense. Niterói, Rio de Janeiro, Brazil

**Keywords:** Hypertension, Kidney Diseases, Mass Screening, Patient Care Team, Renal Insufficiency, Chronic., Hipertensión, Enfermedades Renales, Tamizaje Masivo, Grupo de Atención al Paciente, Insuficiencia Renal Crónica.

## Abstract

**Objectives:**

to analyze factors related to the risk of developing chronic kidney disease using the SCORED questionnaire in resistant hypertensive individuals.

**Methods:**

this cross-sectional study was conducted at a university hospital in Rio de Janeiro state, between January 2022 and October 2024, with an intentional, non-probabilistic sample of 63 participants. Sociodemographic, clinical, and lifestyle data were collected from medical records, and the Screening for Occult Renal Disease (SCORED) questionnaire was applied via telemonitoring to estimate the risk of chronic kidney disease. Fisher’s exact and Mann-Whitney tests were used for statistical analysis.

**Results:**

there was a predominance of females (80.3%), mean age of 66.4 ± 10.0 years, ≥ 21 years since diagnosis of hypertension, and 96.8% at risk for kidney disease.

**Conclusions:**

in this first study with resistant hypertensive patients at risk for kidney disease, the related factors were sex, advanced age and time since clinical diagnosis of arterial hypertension.

## RESUMEN

**Objetivos:**

analizar los factores relacionados con el riesgo de desarrollar enfermedad renal crónica mediante el cuestionario SCORED em individuos hipertensos resistentes.

**Métodos:**

este estudio transversal se realizó em un hospital universitario em el estado de Río de Janeiro, entre enero de 2022 y octubre de 2024, con una muestra intencional, no probabilística de 63 participantes. Se recopilaron datos sociodemográficos, clínicos y de estilo de vida de las historias clínicas, y se aplicó el cuestionario de detección de enfermedad renal oculta (SCORED) mediante telemonitorización para estimar el riesgo de enfermedad renal crónica. Se utilizaron las pruebas exacta de Fisher y Mann-Whitney para el análisis estadístico.

**Resultados:**

hubo un predominio de mujeres (80,3%), edad media de 66,4 ± 10,0 años, ≥ 21 años desde el diagnóstico de hipertensión y 96,8% en riesgo de enfermedad renal.

**Conclusiones:**

en este primer estudio con pacientes hipertensos resistentes con riesgo de enfermedad renal, los factores relacionados fueron sexo, edad avanzada y tiempo desde el diagnóstico clínico de hipertensión arterial.

## INTRODUCTION

According to the World Health Organization (WHO)^(1)^, an estimated 50 million Brazilians have a clinical diagnosis of Systemic Arterial Hypertension (SAH), or Arterial Hypertension (AH). It is a chronic, non-communicable disease characterized by a sustained elevation in blood pressure levels ≥140 mmHg systolic and/or 90 mmHg diastolic^(2)^. Additionally, the classification of SAH includes Resistant Arterial Hypertension (RAH), defined as uncontrolled blood pressure despite the use of three or more antihypertensive medications in adequate doses, including a diuretic, or the use of four or more blood pressure control medications^(2)^.

High blood pressure can be aggravated by the presence of other risk factors such as abdominal obesity, a sedentary lifestyle, dyslipidemia, glucose intolerance, diabetes mellitus, smoking, and excessive alcohol consumption. There is an independent association with events such as stroke (stroke), acute myocardial infarction (AMI), sudden death, heart failure (HF), peripheral arterial disease (PAD), and chronic kidney disease (CKD)^(3)^.

Therefore, hypertension and kidney function have an important relationship, making hypertension both a cause and a consequence of kidney disease^(4)^. It is important to emphasize that treatment for hypertension should be initiated early, as untreated and uncontrolled blood pressure levels can contribute to the progression of chronic kidney disease (CKD).

Hypertensive nephrosclerosis is a progressive kidney disease that causes severe chronic kidney failure and it is the second most common cause of kidney failure in both developed and developing countries^(5)^. In Brazil, in recent years, hypertension has been the main etiological factor associated with CKD^(6)^. There is scientific evidence that the general population is unaware of the relationship between hypertension and the underlying triggering condition in chronic kidney disease. Furthermore, this knowledge is also deficient in the population diagnosed with hypertension, which raises awareness of the need for multidisciplinary interventions for self-management of care^(7)^.

This need is supported by the fact that CKD is a condition with an asymptomatic onset, often characterized by slow and silent progression of renal function loss^(8)^. Furthermore, in more advanced stages of the disease, in addition to a decline in the individual’s quality of life, CKD generates high costs to the healthcare system due to the need for Renal Replacement Therapies (hemodialysis, peritoneal dialysis, and kidney transplant)^(9)^.

It is noteworthy that for screening and assessing the risk of developing CKD in clinical practice, clinical and laboratory examinations are used^(10)^, as well as predictive scores^(11)^, such as the Screening for Occult Renal Disease (SCORED) questionnaire^(12)^, for which one study indicated high sensitivity (97%), specificity of 23%, and accuracy of 47%^(11)^.

Therefore, the scarcity of studies on resistant hypertensive individuals, related factors, and the risk of developing CKD identified by SCORED allows for accessible, early screening in clinical practice and offers the possibility of interventions in prevention, health promotion, and treatment.

## OBJECTIVES

To analyze factors related to the risk of developing chronic kidney disease using the SCORED questionnaire in resistant hypertensive patients.

## METHODS

### Ethical aspects

The study was submitted to and approved by the Research Ethics Committee (REC) of the proposing institution, in accordance with ethical criteria. Participants’ privacy was guaranteed by coding “P001 to P100”. All participants forwarded the invitation and the Informed Consent Form (ICF) to the researchers via WhatsApp, voluntarily accepting to participate in the study. These procedures comply with Resolution 466/2012 of the National Health Council and Circular Letter No. 2/2021/CONEP/SECNS/MS, specifically for virtual research during the COVID-19 pandemic.

### Study design, period and location

This observational, cross-sectional, retrospective, descriptive, and analytical study, with a quantitative approach, was reported according to the Strengthening the Reporting of Observational Studies in Epidemiology (STROBE) guidelines^(13)^. It is an integral part of the “Interdisciplinary Project for Virtual Monitoring of Systemic Arterial Hypertension (PISAV_HAS) in the Context of the COVID-19 Pandemic - Phase I”.

The research was conducted at the Resistant Hypertension Outpatient Clinic of the Antônio Pedro University Hospital (HUAP) of the Fluminense Federal University (UFF), from January 2022 to October 2024.

### Population, inclusion and exclusion criteria

At the aforementioned outpatient clinic, 180 resistant hypertensive patients were registered between January 2022 and October 2024. The inclusion criteria were: age 18 or older and both genders. The exclusion criterion was hospitalization at the time of data collection. Thus, a group composed of faculty, healthcare professionals, undergraduate and graduate students trained for this purpose, and participants in the interdisciplinary project, made five attempts to contact each of the 180 resistant hypertensive patients receiving multidisciplinary outpatient care by telephone. However, due to problems with missed calls, nonexistent numbers, and changes in contact numbers, 90 of them were contacted. Furthermore, during this period, there were 23 losses to follow-up and 4 deaths, resulting in a purposeful, non-probabilistic sample of 63 participants.

### Study protocol

After the invitation, acceptance and signing of the ICF, the first step was to consult the medical records to collect sociodemographic data (gender, age, self-declared skin color, marital status and education), clinical data (dyslipidemia, obesity, time since diagnosis of hypertension and blood pressure value at the last consultation) and lifestyle habits (smoking and alcohol consumption). For this purpose, a form designed and called “Instrument - Data Collection from Clinical Records” was used from January 2022 to October 2024.

In the second stage, to identify the estimated risk of resistant hypertensive patients for developing chronic kidney disease, the SCORED (Screening for Occult Renal Disease) questionnaire was administered via telemonitoring for approximately 20 minutes from March 2022 to October 2024. This questionnaire, translated and validated in Brazil in 2012^(12)^, has 11 questions, each with different scoring options. Age is assigned 2 points for individuals aged 50 to 59, 3 points for those aged 60 to 69, and 4 points for those aged >70. For the remaining questions, 1 point is scored for each positive response by the individual regarding: being female; currently having or currently having anemia; having high blood pressure; being diabetic; a history of heart attack or stroke; congestive heart failure or heart failure; circulation problems/circulatory disease in the legs; and a test showing protein in the urine.

At the end of the questionnaire, all the points for each question answered positively are added up. Therefore, if a person scored 4 or more points, it indicates a 1 in 5 chance of developing chronic kidney disease.

### Analysis of results and statistics

After data collection, statistical analyses were performed using the Statistical Package for Social Sciences (SPSS^®^) software, version 22, and R, version 4.3.1, assuming a significance level of 5%.

Categorical variables were described as absolute frequencies and percentages, while numerical variables were expressed as means and standard deviations. Fisher’s exact test was used to assess the relationship between sociodemographic variables, lifestyle habits, and comorbidities and the risk of chronic kidney disease, as assessed by the SCORED questionnaire. The relationship between the age (in years) of the survey participants and the SCORED score (zero to three points versus four points or more) was assessed using the Mann-Whitney test.

## RESULTS

**Figure 1:**
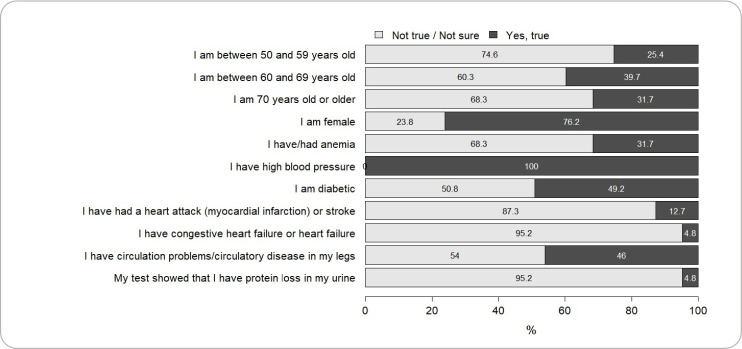
Responses to the Screening for Occult Renal Disease questionnaire, Niterói, Rio de Janeiro, Brazil, 2025

The final sample size was composed by 63 participants. Figure 1 shows a predominance of women (80.3%), with the majority aged 60 to ≥ 70 years (71.4%), 31.7% reporting having or having had anemia, 49.2% reporting diabetes, and 46% reporting circulatory disease or leg circulation problems. Furthermore, only 12.7% reported having had a heart attack or stroke, and 4.8% reported heart failure. Regarding the question “my test showed that I have protein loss in my urine”, only 4.8% responded yes.

*Stroke - Cerebrovascular accident.*

Sociodemographic data, as well as their relationship with the risk or absence of chronic kidney disease, according to the SCORED questionnaire, are presented in Table 1. Thus, it is possible to observe that 96.8% of the research participants were at risk of developing chronic kidney disease.

**Table 1:** Sociodemographic characterization and relationship with total Screening for Occult Renal Disease questionnaire scores, Niterói, Rio de Janeiro, Brazil, 2025

	Total	Total points		*p* value
		0 to 3 n=2 (3.2%)	4 or mores n=61 (96.8%)	
**Gender**				
Female	49 (77.8%)	0 (0%)	49 (80.3%)	0.047^*^†
Male	14 (22.2%)	2 (100%)	12 (19.7%)	
**Age**				
< 50 years	2 (3.2%)	0 (0%)	2 (3.3%)	0.110^*^
50 to59 years	14 (22.2%)	2 (100%)	12 (19.7%)	
60 to 69 years	21 (33.3%)	0 (0%)	21 (34.4%)	
70 years or +	26 (41.3%)	0 (0%)	26 (42.6%)	
**Self-declared skin color**				
White	16 (25.4%)	1 (50%)	15 (24.6%)	1.000^*^
Brown	31 (49.2%)	1 (50%)	30 (49.2%)	
Black	16 (25.4%)	0 (0%)	16 (26.2%)	
**Maritul status**				
In a stable union	29 (46.0%)	0 (0%)	29 (47.5%)	0.287^*^
Without a steady partner	23 (36.5%)	2 (100%)	21 (34.4%)	
Widower	11 (17.5%)	0 (0%)	11 (18.0%)	
**Education**				
Incomplete Elementary	13 (20.6%)	1 (50%)	12 (19.7%)	0.381^*^
Complete Elementary/Incomplete High School	21 (33.3%)	1 (50%)	20 (32.8%)	
Complete High School/Incomplete High School	26 (41.3%)	0 (0%)	26 (42.6%)	
Complete High School	3 (4.8%)	0 (0%)	3 (4.9%)	

* *Fisher's exact test; †p value significant at the 5% level.*

Table 1 shows that the majority of the participants at risk were female (80.3%), thus presenting statistical significance (p=0.047).

Regarding age, the mean age of all participants was 66.4 ± 10.0 years, while those at risk for CKD had a mean age of 66.8 ± 10.0 years, and those not at risk had a mean age of 54.5 ± 5.0 years (data not tabulated). Furthermore, the p-value was not statistically significant (p=0.052), which indicates that the older the participant, the higher the SCORED score. Regarding the other items evaluated (skin color, marital status and education), no statistical significance was evidenced, with the highest prevalence being brown skin color, having a steady partner and having completed secondary education/incomplete higher education.

Regarding comorbidities, lifestyle habits, and time since hypertension diagnosis, Table 2 shows that 47.6% were dyslipidemic and 6.3% were obese. Regarding smoking and alcohol consumption, it was found that resistant hypertensive individuals had never smoked (71.4%), as well as were non-alcoholics (81%).

**Table 2:** Association between clinical data and lifestyle habits and total Screening for Occult Renal Disease questionnaire scores, Niterói, Rio de Janeiro, Brazil, 2025

	Total	Total points		*p* value
		0 to 3 n=2 (3.2%)	4 or mores n=61 (96.8%)	
**Dyslipidemia**				
No	33 (52.4%)	2 (100%)	31 (50.8%)	0.493^*^
Yes	30 (47.6%)	0 (0%)	30 (49.2%)	
**Obesity**				
No	59 (93.7%)	2 (100%)	57 (93.4%)	1.000^*^
Yes	4 (6.3%)	0 (0%)	4 (6.6%)	
**Smoking**				
Never	45 (71.4%)	1 (50.0%)	44 (72.1%)	0.493^*^
Stopped over a year ago	9 (14.3%)	1 (50.0%)	8 (13.1%)	
Yes	5 (7.9%)	0 (0%)	5 (8.2%)	
Not provided	4 (6.3%)	0 (0%)	4 (6.6%)	
**Alcoholism**				
No	51 (81.0%)	2 (100%)	49 (80.3%)	1.000^*^
Yes	12 (19.0%)	0 (0%)	12 (19.7%)	
**Time of HAS diagnosis**				
10 years or younger	13 (20.6%)	0 (0%)	13 (21.3%)	1.000^*^
11 to 20 years	24 (38.1%)	1 (50.0%)	23 (37.7%)	
21 years or older	26 (41.1%)	1 (50.0%)	25 (41.0%)	

HAS - Systemic arterial hypertension;

* Fisher's exact test.

Regarding time since hypertension diagnosis, most participants had been diagnosed with hypertension for 21 years or more (41.1%).

Chart 1 shows the behavior of blood pressure (systolic and diastolic) according to age group. Thus, it is possible to observe that the highest mean systolic pressure for females was in the age group <50 years (155.0 ± 7.1 mmHg), and in males the highest mean systolic pressure was between the ages of 50 and 59 years (149.8 ± 24.3 mmHg). Regarding the diastolic pressure value, the highest mean prevailed in the age group of 50 to 59 years for females (90.7 ± 21.8), and for males it was also for the age group of 50 to 59 years (88.2 ± 13.3 mmHg).

**Chart 1:** Blood pressure behavior according to age group, Niterói, Rio de Janeiro, Brazil, 2025.

	SBP (mmHg)		DBP (mmHg)	
	Female	Male	Female	Male
**Age**				
< 50 years	155.0 ± 7.1	-	90.0 ± 14.1	-
50 to 59 years	153.6 ± 28.3	149.8 ± 24.3	90.7 ± 21.8	88.2 ± 13.3
60 to 69 years	142.3 ± 19.8	137.3 ± 4.6	79.5 ± 11.8	86.0 ± 6.9
70 year sor more	150.1 ± 21.0	145.2 ± 8.2	80.9 ± 12.8	79.7 ± 7.6

*SBP - Systolic blood pressure in mmHg; DBP - Diastolic blood pressure in mmHg.*

## DISCUSSION

In this first screening study for chronic kidney disease in resistant hypertensive individuals, 96.8% were found to be at risk for the disease, based on the Screening for Occult Renal Disease (SCORED) questionnaire^(11)^, representing a promising tool for risk stratification. The application of SCORED to samples of 221^(11)^ and 296^(14)^ hypertensive and/or diabetic individuals demonstrated, respectively, high sensitivity of 97% and 97.1%, specificity of 23% and 14.4%, accuracy of 47%^(11)^, positive predictive value of 27.3%, and negative predictive value of 93.9%^(11)^. This fact reinforces the practicality of SCORED as a screening strategy for chronic kidney disease.

Implementing SCORED in clinical practice can facilitate CKD screening among hypertensive individuals in specialized secondary care, contributing to early detection and appropriate management of the disease. Its simplicity and low cost make it a viable option, especially in resource-limited settings, such as primary health care settings. However, it is important to consider its low specificity as a limitation, which can result in false positives^(11)^. Therefore, the use of SCORED should be complemented by clinical and laboratory evaluations to confirm the diagnosis of kidney disease.

Therefore, after initial risk screening, further clinical and laboratory evaluations are necessary.

CKD is said to be more common in women, and in the sample studied, it was possible to identify that hypertensive women have a higher risk of developing the disease compared to men. However, the disease progresses more slowly in women than in men. Since detection is earlier in women, they tend to seek preventive health services regularly to confirm or rule out kidney disease^(15)^. However, there is a high incidence among men due to their possible delayed access to health services, and consequently, they are in more advanced stages of kidney disease^(16)^. The differences in the prevalence of CKD between men and women are evidenced by the severity of the disease. In the early stages of CKD (stages 1 to 4), women are more prevalent, while in advanced stages, such as dialysis, men are more prevalent^(17)^.

Regarding age, the older the age group, the greater the risk of developing chronic kidney disease. Advanced age is a significant risk factor for the development of chronic kidney disease in hypertensive individuals. With aging, structural changes occur in the kidneys, such as renal atrophy and a reduction of approximately 10% of the renal cortex per decade after the age of 30, compromising renal function^(10)^.

Furthermore, chronic kidney disease is more prevalent in individuals aged 65 and older, especially among those with hypertension. Hypertension, when uncontrolled, can worsen the decline in kidney function in the elderly, making them more likely to develop CKD^(17)^.

Furthermore, mortality from CKD secondary to hypertension is higher in older age groups. Data from Brazil show that individuals aged 70 and older have the highest mortality rates from CKD associated with hypertension. These findings reinforce the importance of multidisciplinary preventive and therapeutic interventions targeting hypertension^(18)^.

Regarding the time since diagnosis of systemic arterial hypertension, there is a risk of developing chronic kidney disease. The literature has shown that prolonged uncontrolled hypertension significantly increases cumulative kidney damage due to the constant hemodynamic overload on the renal glomeruli. Over time, this elevated pressure promotes irreversible structural changes, such as glomerular sclerosis and reduced glomerular filtration, contributing to the progression of CKD^(19)^.

Individuals with hypertension for more than 10 years have a higher prevalence of microalbuminuria, an early marker of kidney damage. This relationship is exacerbated by the presence of other risk factors, such as diabetes and obesity. However, the duration of exposure to high blood pressure is one of the main determinants of renal decline^(20)^. In this study, it was observed that the majority of the population at risk for CKD had been diagnosed with hypertension for more than 21 years.

Furthermore, the relationship between the time since hypertension diagnosis and the risk of progression to end-stage renal disease (ESRD) was observed in individuals diagnosed with hypertension for more than 20 years. This effect was more pronounced in patients who did not adhere to treatment or experienced significant variations in blood pressure over time. The study reinforces that the duration of exposure to hypertension plays a critical role in renal impairment, highlighting the importance of specific and sustained blood pressure control at all stages of hypertension. Given the comorbidities of diabetes and obesity, it is important to remember that these conditions increase the risk of developing diseases such as CKD. Obesity contributes to the progression of kidney disease stages through hyperfiltration to meet the demands of body weight and increased intraglomerular pressure, which damages renal structures^(19)^. However, a low prevalence of obesity was identified in this sample. However, 47.6% were dyslipidemic, for which the literature has shown that patients with chronic kidney disease have different lipid profiles depending on the stages of the disease and their treatment options. Among chronic kidney patients undergoing conservative treatment, the main disorder found was hypertriglyceridemia^(21)^.

Finally, blood pressure (systolic and diastolic) behavior was analyzed for both genders, by age group, given the importance of monitoring and controlling blood pressure values.

### Study limitations

The cross-sectional study design with a purposive, non-probabilistic sample requires caution regarding the extrapolation of results and reproducibility. Therefore, future studies with different study designs and sample sizes are suggested.

Another limitation is the difficulty researchers have in contacting eligible participants by phone, due to barriers such as missed calls, nonexistent phone numbers, and changes in contact numbers.

### Contributions to the field of Nursing

The use of SCORED is considered relevant for estimating the risk of developing chronic kidney disease in resistant hypertensive patients^(11)^. Its application by nurses in clinical practice for early identification of the disease contributes to the planning of interventions and the adoption of preventive strategies^(22)^. Furthermore, since it is possible for nurses to apply the SCORED questionnaire during telemonitoring, as an innovative action, it can expand the reach and benefits of professional health care.

## CONCLUSIONS

Using the Screening for Occult Renal Disease (SCORED), we analyzed the factors related to the risk of developing chronic kidney disease in 63 resistant hypertensive patients, of whom 96.8%, as estimated, were at risk of developing kidney disease. To this end, we investigated the association between gender, age, self-reported skin color, marital status, education, dyslipidemia, obesity, smoking, alcohol consumption, and time since hypertension diagnosis. We also analyzed the behavior of diastolic and systolic blood pressure between male and female genders.

This observational study showed that the most significant related factors in this sample, which require planning for multidisciplinary interventions in individualized care, were female gender, advanced age, and prolonged time since hypertension diagnosis.

## Data Availability

The research data are available within the article.
