# Supplementary material for: Factors related to the risk of developing chronic kidney disease in resistant hypertensive patients: a cross-sectional study
Source: Rev Bras Enferm. 2026 Jun 12;79:e20250160. doi: 10.1590/0034-7167-2025-0160 (PMC13262983; doi:10.1590/0034-7167-2025-0160)
Supplement: Translation

# Translation: Fatores relacionados ao risco de desenvolvimento da doença renal crônica em hipertensos resistentes: estudo transversal

**DOI:** 10.1590/0034-7167-2025-0160pt

**Authors:** Alessandra de Oliveira Guimarães, Nicole Oliveira Santana, Ronaldo Altenburg Gismondi, Dayse Mary da Silva Correia

**Affiliations:** I Universidade Federal Fluminense. Niterói, Rio de Janeiro, Brasil

**Keywords:** Hipertensão, Nefropatias, Programas de Rastreamento, Equipe de Assistência ao Paciente, Insuficiência Renal Crônica.

## Abstract

**Objetivos:**

analisar os fatores relacionados ao risco de desenvolvimento da doença renal crônica por meio do questionário SCORED em hipertensos resistentes.

**Métodos:**

estudo transversal, realizado em hospital universitário fluminense, entre janeiro de 2022 a outubro de 2024, com amostra intencional, não probabilística de 63 participantes. No prontuário, houve a coleta de dados sociodemográficos, clínicos e hábitos de vida, e via telemonitoramento, aplicou-se o *Screening For Occult Renal Disease*(SCORED) para estimativa de risco da doença renal crônica. Na análise estatística, utilizou-se os testes Exato de *Fisher* e *Mann-Whitney*.

**Resultados:**

predominância feminina (80,3%), idade média de 66,4 ± 10,0 anos, ≥ 21 anos de diagnóstico de hipertensão arterial e 96,8% com risco para doença renal.

**Conclusões:**

nesse primeiro estudo com hipertensos resistentes com risco para a doença renal, os fatores relacionados foram o sexo, a idade avançada e o tempo do diagnóstico clínico de hipertensão arterial.

## INTRODUÇÃO

Segundo o Relatório da Organização Mundial de Saúde (OMS)^(1)^, estima-se que 50 milhões de brasileiros tenham o diagnóstico clínico de Hipertensão Arterial Sistêmica (HAS), ou Hipertensão Arterial (HA). É uma doença crônica não transmissível caracterizada por elevação sustentada nos níveis de pressão arterial ≥ 140 mmHg para sistólica e/ou 90 mmHg para diastólica^(2)^. Além disso, há na classificação da HAS a Hipertensão Arterial Resistente (HAR), definida pela pressão arterial não controlada apesar do uso de três ou mais medicamentos anti-hipertensivos em doses adequadas, incluindo um diurético, ou, ainda, decorrente do uso de quatro ou mais medicamentos para o controle da pressão arterial^(2)^.

A hipertensão arterial pode ser agravada pela presença de outros fatores de risco como obesidade abdominal, sedentarismo, dislipidemia, intolerância à glicose, diabetes mellitus, tabagismo e excesso de bebida alcoólica. Havendo associação independente com eventos como o Acidente Vascular Encefálico (AVE), Infarto Agudo do Miocárdio (IAM), morte súbita, Insuficiência Cardíaca (IC), Doença Arterial Periférica (DAP) e Doença Renal Crônica (DRC)^(3)^.

Portanto, a hipertensão arterial e a função renal têm uma relação importante, o que faz com que a hipertensão seja tanto causa como consequência da doença renal^(4)^. Cabe enfatizar que o tratamento da HAS deve ser iniciado de modo precoce, visto que o não tratamento e a falta de controle dos níveis pressóricos podem contribuir para evolução da doença renal crônica (DRC).

A nefroesclerose hipertensiva é uma doença renal progressiva causadora de insuficiência renal crônica grave, sendo a segunda causa mais frequente de insuficiência renal tanto nos países desenvolvidos como nos países em desenvolvimento^(5)^. E, no Brasil, nos últimos anos, a hipertensão foi o principal fator etiológico relacionado com a DRC^(6)^.

Há evidências científicasde que a população desconhece a relação da hipertensão como patologia de base desencadeante da doença renal crônica. Ainda, na população com diagnóstico de HAS esse conhecimento também é deficiente, o que aumenta o alerta sobre a necessidade de intervenções multiprofissionais para a autogestão do cuidado^(7)^.

Tal necessidade apoia-se no fato da DRC ser uma patologia de início assintomático, na maioria das vezes, com característica de progressão lenta e silenciosa da perda da função renal^(8)^. Ademais, quando em fases mais avançadas da doença, além da queda na qualidade de vida do indivíduo, a DRC gera um alto custo ao sistema de saúde no que tange da necessidade de Terapias Renais Substitutivas (hemodiálise, diálise peritoneal e transplante renal)^(9)^.

Destaca-se que, para o rastreamento e avaliação do risco de desenvolvimento de DRC na prática clínica, há o exame clínico e laboratorial^(10)^, bem como scores preditores^(11)^,como o questionário Screening For Occult Renal Disease(SCORED)^(12)^, para o qual um estudo indicou alta sensibilidade (97%), especificidade de 23%, com uma acurácia de 47%^(11)^.

Portanto, a escassez de estudos com hipertensos resistentes, com fatores relacionados e o risco de desenvolvimento da DRC identificado pelo SCORED permite, na prática clínica, um rastreamento acessível, precoce e traz possibilidade para intervenções de prevenção, promoção e tratamento em saúde.

## OBJETIVOS

Analisar os fatores relacionados ao risco de desenvolvimento da doença renal crônica por meio do questionário SCORED em hipertensos resistentes.

## MÉTODOS

### Aspectos éticos

O estudo foi submetido e aprovado pelo Comitê de Ética e Pesquisa (CEP) da instituição proponente, obedecendo aos critérios éticos. Quanto à privacidade dos participantes, foi garantido o anonimato por meio da codificação “P001 a P100”, todos os participantes reencaminharam aos pesquisadores, via *WhatsApp*, o convite e o Termo de Consentimento Livre e Esclarecido (TCLE), com o aceite voluntário para participação da pesquisa. Tais procedimentos estão em conformidade com a Resolução 466/2012 do Conselho Nacional de Saúde e do Ofício Circular nº 2/2021/CONEP/SECNS/MS, específico para pesquisas em ambiente virtual durante a pandemia de COVID-19.

### Desenho, período e lo+cal do estudo

Estudo observacional, transversal, retrospectivo, descritivo e analítico, com abordagem quantitativa, reportado segundo as diretrizes do *Strengthening the Reporting of Observational studies in Epidemiology* (STROBE)^(13)^. É parte integrante do “Projeto Interdisciplinar para Seguimento em Ambiente Virtual da Hipertensão Arterial Sistêmica (PISAV_HAS) no Contexto da Pandemia de COVID-19 - Fase I”.

A pesquisa foi desenvolvida no Ambulatório de Hipertensão Resistente do Hospital Universitário Antônio Pedro (HUAP) da Universidade Federal Fluminense (UFF), no período de janeiro de 2022 a outubro de 2024.

### População, critérios de inclusão e exclusão

No citado ambulatório, havia 180 hipertensos resistentes cadastrados, entre janeiro de 2022 e outubro de 2024. Os critérios de inclusão estabelecidos foram: idade maior ou igual a 18 anos de ambos os sexos. Por outro lado, o critério de exclusão foi a hospitalização no momento da coleta dos dados. Por meio de um grupo composto por docentes, profissionais de saúde, discentes de graduação e pós-graduação treinados para essa finalidade e participantes do projeto interdisciplinar, houve cinco tentativas de contato telefônico a cada um dos 180 hipertensos resistentes sob atendimento ambulatorial multiprofissional. Entretanto, por problemas com chamadas não atendidas, números inexistentes e mudança de número de contato, foram contactadas 90 pessoas. Além disso, no período, houve 23 perdas de seguimento, 4 óbitos, compondo uma amostra intencional, não probabilística, de 63 participantes.

### Protocolo do estudo

Após o convite, aceite e assinatura do TCLE houve, como primeira etapa, a consulta ao prontuário para coleta de dados sociodemográficos (sexo, idade, cor da pele autodeclarada, estado civil e escolaridade), clínicos (dislipidemia, obesidade, tempo de diagnóstico de HAS e valor de pressão arterial em última consulta) e hábitos de vida (tabagismo e etilismo). Para tal finalidade, foi utilizado um formulário elaborado e denominado de “Instrumento - Coleta de Dados em Prontuário Clínico”, no período de janeiro de 2022 a outubro de 2024.

Na segunda etapa, no intuito de identificação da estimativa de risco dos hipertensos resistentes para o desenvolvimento da doença renal crônica, aplicou-se por telemonitoramento, durante aproximadamente 20 minutos, o questionário SCORED (*Screening For Occult Renal Disease*), no período de março de 2022 a outubro de 2024. Tal questionário, traduzido e validado no Brasil no ano de 2012^(12)^, possui 11 perguntas, para as quais há pontuações diferentes, de modo que, para a idade, atribui-se 2 pontos para indivíduos de 50 a 59 anos, 3 pontos para os de 60 a 69 anos e 4 pontos para >70 anos. Nas demais perguntas, marca-se 1 ponto para cada resposta positiva do indivíduo: ser do sexo feminino; já ter histórico prévio ou atual de anemia; ter pressão alta; ser diabético; possuir histórico de ataque cardíaco (infarto) ou derrame/AVC/AVE; ter insuficiência cardíaca congestiva ou insuficiência cardíaca; ter problema de circulação/doença circulatória nas pernas e ter exame que mostra presença de proteína na urina.

Ao final da aplicação do questionário, somam-se todos os pontos de cada pergunta respondida positivamente. Portanto, caso a pessoa tenha obtido 4 ou mais pontos, indica-se que há 1 chance em 5 de desenvolver a doença renal crônica.

### Análise dos resultados e estatística

Após a coleta de dados, as análises estatísticas foram realizadas nos *Softwares Statistical Package for Social Sciences* (SPSS^®^), versão 22 e R, versão 4.3.1, considerando um nível de significância de 5%.

As variáveis categóricas foram descritas através de frequências absolutas e percentuais, enquanto as numéricas foram expressas por médias e desvios padrões. O teste exato de *Fisher* foi utilizado para avaliar a relação entre variáveis sociodemográficas, hábitos de vida, comorbidades e o risco para doença renal crônica, avaliado pelo questionário SCORED. A relação entre a idade (em anos) dos participantes da pesquisa e a pontuação do SCORED (zero a três pontos versus quatro pontos ou mais) foi avaliada através do teste de *Mann-Whitney*.

## RESULTADOS

O n final da amostra foi de 63 participantes. Na Figura 1, observa-se a predominância feminina (80,3%), a maioria com idade entre 60 a ≥ 70 anos (71,4%), 31,7% afirmaram que têm ou já tiveram anemia, 49,2% referiram serem diabéticos e 46% com doença circulatória ou problema de circulação nas pernas. E ainda, apenas 12,7% responderam que já tiveram ataque cardíaco (infarto) ou derrame/AVC/AVE, e 4,8% relataram ter insuficiência cardíaca. Quanto à pergunta “meu exame mostrou que eu tenho perda de proteína na minha urina” apenas 4,8% responderam que sim.

**Figura 1:** Respostas ao questionário Screening For Occult Renal Disease, Niterói, Rio de Janeiro, Brasil, 2025

*AVC - Acidente vascular cerebral; AVE - Acidente vascular encefálico.*

Os dados sociodemográficos, bem como a sua relação com o risco ou não para doença renal crônica, conforme o questionário SCORED, são apresentados na Tabela 1. Dessa forma, é possível observar que 96,8% dos participantes da pesquisa apresentaram risco para desenvolver doença renal crônica.

**Tabela 1:** Caracterização sociodemográfica e relação com o total de pontos do questionário *Screening For Occult Renal Disease*, Niterói, Rio de Janeiro, Brasil, 2025

	Total	Total de pontos		Valor de *p*
		0 a 3 n=2 (3,2%)	4 ou mais n=61 (96,8%)	
**Sexo**				
Feminino	49 (77,8%)	0 (0%)	49 (80,3%)	0,047^*^†
Masculino	14 (22,2%)	2 (100%)	12 (19,7%)	
**Idade**				
< 50 anos	2 (3,2%)	0 (0%)	2 (3,3%)	0,110^*^
50 a 59 anos	14 (22,2%)	2 (100%)	12 (19,7%)	
60 a 69 anos	21 (33,3%)	0 (0%)	21 (34,4%)	
70 anos ou +	26 (41,3%)	0 (0%)	26 (42,6%)	
**Cor da pele autodeclarada**				
Branca	16 (25,4%)	1 (50%)	15 (24,6%)	1,000^*^
Parda	31 (49,2%)	1 (50%)	30 (49,2%)	
Preta	16 (25,4%)	0 (0%)	16 (26,2%)	
**Estado civil**				
Com companheiro(a) fixo	29 (46,0%)	0 (0%)	29 (47,5%)	0,287^*^
Sem companheiro(a) fixo	23 (36,5%)	2 (100%)	21 (34,4%)	
Viúvo(a)	11 (17,5%)	0 (0%)	11 (18,0%)	
**Escolaridade**				
Fundamental Incompleto	13 (20,6%)	1 (50%)	12 (19,7%)	0,381^*^
Fundamental Completo/Médio Incompleto	21 (33,3%)	1 (50%)	20 (32,8%)	
Médio Completo/Superior Incompleto	26 (41,3%)	0 (0%)	26 (42,6%)	
Superior Completo	3 (4,8%)	0 (0%)	3 (4,9%)	

* *Teste exato de Fisher; †Valor de p significativo ao nível de 5%.*

A Tabela 1 mostra que a maior parte dos participantes com risco é do sexo feminino (80,3%), apresentando, assim, significância estatística (p= 0,047).

Quanto à idade, a média do total de participantes foi de 66,4 ± 10,0 anos, enquanto para aqueles que apresentaram risco para DRC, a média da idade foi de 66,8 ± 10,0 anos, os que não apresentaram risco tiveram uma média de 54,5 ± 5,0 anos (dados não tabulados). Além disso, o p-valor não foi estatisticamente significativo (p= 0,052), nota-se que quanto maior a idade, maior é a pontuação no SCORED.

Em relação aos demais itens avaliados (cor da pele, estado civil e escolaridade), não foi evidenciada significância estatística, tendo como maior prevalência a cor parda, com companheiro(a) fixo e com ensino médio completo/superior incompleto.

Quanto às comorbidades, hábitos de vida e tempo de diagnóstico da HAS, a Tabela 2 mostra que 47,6% eram dislipidêmicos e 6,3% eram obesos. Para o tabagismo e etilismo, identificou-se que os hipertensos resistentes nunca haviam fumado (71,4%), bem como não eram etilistas (81%).

**Tabela 2:** Associação entre dados clínicos e hábitos de vida e o total de pontos ao questionário *Screening For Occult Renal Disease*, Niterói, Rio de Janeiro, Brasil, 2025

	Total	Total de pontos		Valor de *p*
		0 a 3 n=2 (3,2%)	4 ou mais n=61 (96,8%)	
**Dislipidemia**				
Não	33 (52,4%)	2 (100%)	31 (50,8%)	0,493^*^
Sim	30 (47,6%)	0 (0%)	30 (49,2%)	
**Obesidade**				
Não	59 (93,7%)	2 (100%)	57 (93,4%)	1,000^*^
Sim	4 (6,3%)	0 (0%)	4 (6,6%)	
**Tabagismo**				
Nunca	45 (71,4%)	1 (50,0%)	44 (72,1%)	0,493^*^
Parou há mais de 1 ano	9 (14,3%)	1 (50,0%)	8 (13,1%)	
Sim	5 (7,9%)	0 (0%)	5 (8,2%)	
Não informado	4 (6,3%)	0 (0%)	4 (6,6%)	
**Etilismo**				
Não	51 (81,0%)	2 (100%)	49 (80,3%)	1,000^*^
Sim	12 (19,0%)	0 (0%)	12 (19,7%)	
**Tempo de diagnóstico HAS**				
10 anos ou menos	13 (20,6%)	0 (0%)	13 (21,3%)	1,000^*^
11 a 20 anos	24 (38,1%)	1 (50,0%)	23 (37,7%)	
21 anos ou mais	26 (41,1%)	1 (50,0%)	25 (41,0%)	

HAS - Hipertensão arterial sistêmica;

* Teste exato de Fisher.

Para o tempo de diagnóstico de HAS, a maior parte dos participantes tinha 21 anos ou mais de tempo de diagnóstico de HAS (41,1%).

O Quadro 1 mostra comportamento da pressão arterial (sistólica e diastólica) conforme a faixa etária. Dessa forma, é possível observar que a maior média de pressão sistólica para o sexo feminino foi na faixa <50 anos (155,0 ± 7,1 mmHg). Já no sexo masculino, a maior média de sistólica foi entre as idades de 50 a 59 anos (149,8 ± 24,3 mmHg). Quanto ao valor de pressão diastólica, prevaleceu a maior média na faixa etária de 50 a 59 anos para o sexo feminino (90,7 ± 21,8), e para o sexo masculino também foi para idade de 50 a 59 anos (88,2 ± 13,3 mmHg).

**Quadro 1:** Comportamento da pressão arterial de acordo com a faixa etária, Niterói, Rio de Janeiro, Brasil, 2025

	PAS (mmHg)		PAD (mmHg)	
	Feminino	Masculino	Feminino	Masculino
**Idade**				
< 50 anos	155,0 ± 7,1	-	90,0 ± 14,1	-
50 a 59 anos	153,6 ± 28,3	149,8 ± 24,3	90,7 ± 21,8	88,2 ± 13,3
60 a 69 anos	142,3 ± 19,8	137,3 ± 4,6	79,5 ± 11,8	86,0 ± 6,9
70 anos ou +	150,1 ± 21,0	145,2 ± 8,2	80,9 ± 12,8	79,7 ± 7,6

*PAS - Pressão arterial sistólica em mmHg; PAD - Pressão arterial diastólica em mmHg.*

## DISCUSSÃO

Nesse primeiro estudo de rastreamento para a doença renal crônica com hipertensos resistentes, identificou-se que 96,8% apresentaram risco para a doença, a partir do questionário *Screening for Occult Renal Disease*(SCORED)^(11)^, sendo uma ferramenta promissora para estratificação do risco. A aplicação do SCORED, em amostras de 221^(11)^ e 296^(14)^ hipertensos e∕ou diabéticos, demonstrou respectivamente alta sensibilidade de 97% e 97,1%, especificidade de 23%e 14,4%,acurácia de 47%^(11)^, valor preditivo positivo de 27,3%, e valor preditivo negativo de 93,9%^(11)^. Tal fato reforça a praticidade do SCORED como uma estratégia de rastreamento para a doença renal crônica.

A implementação do SCORED na prática clínica pode facilitar o rastreamento da DRC entre hipertensos na atenção secundária especializada, contribuindo para a detecção precoce e manejo adequado da doença. A simplicidade e baixo custo tornam o instrumento uma opção viável, especialmente em áreas com recursos limitados, como em contextos de atenção primária à saúde. Entretanto, é importante que seja considerado como limitação a baixa especificidade, que poderá resultar em falsos positivos^(11)^. Portanto, o uso do SCORED deve ser complementado por avaliações clínicas e laboratoriais para confirmar o diagnóstico da doença renal.

Daí, após o rastreamento inicial do risco, faz-se necessário a complementação por avaliações clínicas e laboratoriais.

A DRC é dita como mais comum em mulheres e, na amostra estudada, foi possível identificar que mulheres hipertensas têm maior risco de desenvolver a doença em comparação ao sexo masculino, embora, a doença progrida mais lentamente no sexo feminino em relação masculino. Nesse sentido, a detecção é mais precoce em mulheres, as quais tendem procurar serviços de prevenção em saúde regularmente para confirmação ou não da doença renal^(15)^.

No sexo masculino, por outro lado, há elevada incidência devido à possível procura tardia dos homens pelos serviços de saúde e, consequentemente, eles se encontram em estágios mais avançados da doença renal^(16)^. As diferenças na prevalência da DRC entre o sexo masculino e feminino se evidenciam pela gravidade da doença, de forma que em estágios iniciais da DRC (1 a 4), há predominância nas mulheres, enquanto em estágios avançados, como a fase dialítica, há predomínio dos homens^(17)^.

No que tange à idade, quanto maior a faixa etária, maior é o risco para desenvolvimento da doença renal crônica. A idade avançada é um fator de risco significativo para o desenvolvimento de doença renal crônica em indivíduos hipertensos. Com o envelhecimento, ocorrem alterações estruturais nos rins, como atrofia renal e redução de aproximadamente 10% do córtex renal por década a partir dos 30 anos, comprometendo a função renal^(10)^.

Ainda, sendo a doença renal crônica mais prevalente em indivíduos com 65 anos ou mais, especialmente entre aqueles com HAS, a hipertensão, quando não controlada, pode piorar o declínio da função renal em idosos, tornando-os mais propensos a desenvolver DRC^(17)^.

Além disso, a mortalidade por DRC secundária à hipertensão é mais elevada em faixas etárias mais avançadas. Dados do Brasil mostram que indivíduos com 70 anos ou mais apresentam as maiores taxas de mortalidade por DRC associadas à hipertensão. Esses achados reforçam a importância de intervenções multiprofissionais preventivas e terapêuticas direcionadas à hipertensão^(18)^.

Em relação ao tempo de diagnóstico de hipertensão arterial sistêmica, há o risco de desenvolvimento de doença renal crônica. Na literatura, tem sido demonstrado que a duração prolongada da hipertensão não controlada aumenta significativamente o dano renal cumulativo, devido à sobrecarga hemodinâmica constante nos glomérulos renais. Com o passar do tempo, essa pressão elevada promove alterações estruturais irreversíveis, como esclerose glomerular e redução da filtração glomerular, contribuindo para a progressão da DRC^(19)^.

Em indivíduos com hipertensão por mais de 10 anos, encontra-se maior prevalência de microalbuminúria, um marcador precoce de lesão renal. Essa relação é exacerbada pela presença de outros fatores de risco, como diabetes e obesidade. Porém o tempo de exposição à pressão arterial elevada é um dos principais determinantes do declínio renal^(20)^. Na presente pesquisa, observou-se que a maioria da população em risco para DRC possuía tempo de diagnóstico de HAS maior que 21 anos.

Ademais, tem-se que a relação entre o tempo de diagnóstico de hipertensão e o risco de progressão para doença renal terminal (DRT) em indivíduos com mais de 20 anos de diagnóstico de hipertensão arterial apresenta um risco 1,8 vezes maior de evolução da doença em comparação com aqueles com menos de cinco anos. Esse efeito foi mais pronunciado em pacientes que não aderiram ao tratamento ou variações significativas na pressão arterial ao longo do tempo. O estudo reforça que o tempo de exposição à hipertensão desempenha um papel crítico na restrição renal, destacando a importância do controle específico sustentado da pressão arterial em todos os estágios da hipertensão.

Mediante as comorbidades diabetes e obesidade, cabe lembrar que as referidas condições elevam o risco da ocorrência de doenças, como a DRC. A obesidade age na progressão dos estágios da doença renal por meio da hiperfiltração, a fim de atender às demandas do peso corporal, e da elevação da pressão intraglomerular, fato que prejudica as estruturas renais^(19)^. Porém, nessa amostra, houve a identificação de baixa prevalência de obesos. Entretanto, 47,6% eram dislipidêmicos, para os quais evidenciou-se na literatura que pacientes com a doença renal crônica possuem perfil lipídico diferente entre os estágios da patologia e suas formas de tratamento. Entre os doentes renais crônicos em tratamento conservador o principal distúrbio encontrado foi a hipertrigliceridemia^(21)^.

Por fim, buscou-se pelo comportamento da pressão arterial (sistólica e diastólica) de ambos os sexos, por faixa etária, dada a importância do acompanhamento e controle dos valores pressóricos.

### Limitações do estudo

O desenho do estudo transversal com uma amostra intencional, não probabilística, infere cautela para extrapolação de resultados e reprodutibilidade. Portanto, sugere-se estudos futuros com diferentes tipos de estudo e cálculo amostral.

Outra limitação encontra-se apoiada na dificuldade dos pesquisadores no contato telefônico com os participantes elegíveis, devido a barreiras como chamadas não atendidas, números inexistentes e mudança do número de contato.

### Contribuições para a área da Enfermagem

Considera-se relevante o uso do SCORED para a estimativa do risco do desenvolvimento da doença renal crônica em hipertensos resistentes^(11)^, considerando sua aplicação por enfermeiros na prática clínica para identificação precoce da doença traz uma contribuição no planejamento de intervençõese a adoção de estratégias preventivas^(22)^. Além disso, é possível a aplicação do questionário SCORED por enfermeiros durante o telemonitoramento, como ação inovadora, pode-se ampliar o alcance e os benefícios do atendimento profissional em saúde.

## CONCLUSÕES

Por meio do *Screening for Occult Renal Disease* (SCORED) foi realizada a análise dos fatores relacionados ao risco de desenvolvimento da doença renal crônica em 63 hipertensos resistentes, dos quais 96,8%, conforme estimado, possuíam o risco de desenvolvimento da doença renal. Para tal, buscou-se pela associação do sexo, idade, cor da pele autodeclarada, estado civil, escolaridade, dislipidemia, obesidade, tabagismo, etilismo, tempo de diagnóstico da hipertensão arterial. Ainda, buscou-se a verificação do comportamento da pressão arterial diastólica e sistólica entre os sexos, feminino e masculino.

Este estudo observacional evidenciou que os fatores relacionados com maior significância na amostra, os quais necessitam de planejamento para intervenções multiprofissionais no cuidado individualizado, deu-se pelo sexo feminino, idade avançada e tempo prolongado do diagnóstico da hipertensão arterial.

## Data Availability

Os dados de pesquisa estão disponíveis no corpo do artigo.
